# A nomogram to predict hyperkalemia in patients with hemodialysis: a retrospective cohort study

**DOI:** 10.1186/s12882-022-02976-4

**Published:** 2022-11-01

**Authors:** Ziwei Mei, Jun Chen, Peipei Chen, Songmei Luo, Lie Jin, Limei Zhou

**Affiliations:** 1 Lishui Municipal Central Hospital, the Fifth Affiliated Hospital of Wenzhou Medical University, Lishui, 323000 Zhejiang China; 2 Zhejiang Chinese Medical University, Hangzhou, 310000 Zhejiang China

**Keywords:** Hemodialysis, Hyperkalemia, Nomogram, Prediction model

## Abstract

**Background:**

Hyperkalemia increases the risk of mortality and cardiovascular-related hospitalizations in patients with hemodialysis. Predictors of hyperkalemia are yet to be identified. We aimed at developing a nomogram able to predict hyperkalemia in patients with hemodialysis.

**Methods:**

We retrospectively screened patients with end-stage renal disease (ESRD) who had regularly received hemodialysis between Jan 1, 2017, and Aug 31, 2021, at Lishui municipal central hospital in China. The outcome for the nomogram was hyperkalemia, defined as serum potassium [K^+^] ≥ 5.5 mmol/L. Data were collected from hemodialysis management system. Least Absolute Shrinkage Selection Operator (LASSO) analysis selected predictors preliminarily. A prediction model was constructed by multivariate logistic regression and presented as a nomogram. The performance of nomogram was measured by the receiver operating characteristic (ROC) curve, calibration diagram, and decision curve analysis (DCA). This model was validated internally by calculating the performance on a validation cohort.

**Results:**

A total of 401 patients were enrolled in this study. 159 (39.65%) patients were hyperkalemia. All participants were divided into development (*n* = 256) and validation (*n* = 145) cohorts randomly. Predictors in this nomogram were the number of hemodialysis session, blood urea nitrogen (BUN), serum sodium, serum calcium, serum phosphorus, and diabetes. The ROC curve of the training set was 0.82 (95%CI 0.77, 0.88). Similar ROC curve was achieved at validation set 0.81 (0.74, 0.88). The calibration curve demonstrated that the prediction outcome was correlated with the observed outcome.

**Conclusion:**

This nomogram helps clinicians in predicting the risk of PEW and managing serum potassium in the patients with hemodialysis.

## Introduction

Hemodialysis is the most common kidney replacement treatment (KRT) used in patients with ESRD. It approximately accounts for 89% of all dialysis [1–3]. In 2018, a survey covering 182 countries’ populations reported that the median use of hemodialysis was 298.4 per million populations [4]. However, hemodialysis is associated with some comorbidities including infection, hyperkalemia, and cardiovascular disease. Hyperkalemia is a highly prevalent complication and a potentially life-threatening electrolyte disorder among populations with hemodialysis [5, 6].

Hyperkalemia is an electrolyte abnormality, clinically characterized as an elevation of serum potassium concentration (>5.5 mmol/L) [7]. This complication often induces symptoms such as nausea, fatigue, and muscle weakness, and severe outcomes in cardiac physiology including chest pain, cardiac dysrhythmia, cardiac arrest, and death [8, 9]. 80–90% of potassium is excreted through kidney. The impairment of kidney on excretory function decreases potassium clearance from human body. This reduction in potassium elimination increases the risk of hyperkalemia [10, 11]. The majority of ESRD patients with hemodialysis have little or no residual renal function. Their potassium clearance almost is dependent on hemodialysis. It is reported that the incidence of hyperkalemia is 25–30% in patients with hemodialysis. Serum potassium of more than 5.6 mmol/L increases all-cause mortality risk by 32%. Compared to hemodialysis patients with normal potassium levels, patients with hyperkalemia are related to a higher risk of all-cause and cardiovascular death [12–15]. Hyperkalemia-related mortality approximately accounts for 4% (range: 0–12.5%) in patients with hemodialysis [5]. Therefore, hyperkalemia prevention places a vital role in the prognosis of patients with hemodialysis. Diuretics reduce serum potassium concentration by accelerating urine excretion. However, diuretic treatment is ineffective in patients with hemodialysis since the majority of them have no urine [16]. Earlier prediction is essential to prevent hyperkalemia in patients with hemodialysis. Several strategies aim to prevent or manage potassium accumulation in patients with hemodialysis. However, there is no evidence-based practice guideline. The predictive model of hyperkalemia in patients with hemodialysis is not quite established. Therefore, it is necessary to develop a model for clinicians in the prediction of hyperkalemia. According to this model, clinicians achieve an individual probability of hyperkalemia occurrence and make interventions.

Nowadays, a nomogram as a visually predictive tool calculates the risk of outcomes for individuals. This tool provides valuable guidance for clinical decision-making. It has been widely applied to evaluate the prognosis of patients recently [17–21]. We aimed to assess risk factors of hyperkalemia and develop a predictive nomogram for patients with hemodialysis.

## Methods

### Study design and participants

We established and validated a nomogram on hyperkalemia prediction in patients with hemodialysis through a retrospective study. All data came from a hemodialysis center in China.

We screened patients treated with maintenance hemodialysis between Jan 1, 2017, and Aug 31, 2021, at a hemodialysis center of Lishui municipal central hospital in China. Eligible participants were over 18 years old. They received continuous hemodialysis for at least 3 months and had regular follow-up visits with vital medical data records. The dialysate potassium concentration in this study was 2 mEq/L. All participants were treated with the same dialysate. The exclusion criteria included unavailable medical information, changed dialysis modality, and trauma/infection occurrence unrelated to hemodialysis within one month.

### Procedure

We randomly divided the whole dataset into training and validation sets with a proportion of 6:4. Thirty-four parameters were collected from the hemodialysis management system. Parameters included basic characteristics, dialysis-related data, and blood laboratory tests. Basic characteristics contained gender, age, height, weight, primary disease, occupation, education, administration history of furosemide or renin-angiotensin-aldosterone system inhibitors (RAASi), and comorbid hypertension or diabetes. Dialysis-related data and blood laboratory tests examined in the last pre-dialysis were collected. Dialysis-related data consisted of blood flow, ultrafiltration volume, hemodialysis vascular access, hemodialysis modality, hemodialysis duration, and the number of hemodialysis sessions. The blood laboratory tests covered serum potassium, hemoglobin, C-reactive protein (CRP), BUN, uric acid, estimated glomerular filtration rate (eGFR), glucose, serum creatinine, serum sodium, serum calcium, albumin, serum phosphorus, triglyceride, total cholesterol, low-density lipoprotein (LDL), high-density lipoprotein (HDL), and parathyroid hormone (PTH). In this study, serum potassium was a single measurement as the endpoint. The serum potassium was measured before the last hemodialysis session of the week. Hyperkalemia was defined as serum potassium [K^+^] ≥ 5.5 mmol/L.

### Statistical analysis

Descriptive statistics contained continuous and categorical variables. The normal distribution of continuous variables was presented by mean + SD. The abnormal distribution was described by median (IQR). Group comparisons of continuous variables were analyzed by T-test or Wilcoxon rank-sum test. Categorical variables were compared by the chi-square test or Fisher’s exact test. Firstly, predictors were preliminarily screened by LASSO regression in the development set. LASSO analysis shrunk the regression coefficient of variables to zero by a penalized coefficient of Lambda. It excluded variables with zero regression coefficients and selected variables without zero regression coefficients. These selected variables were viewed as the most correlated with hyperkalemia. They were presented as odds ratio (OR) with 95% confidence intervals (CIs). Secondly, multivariate logistic regression analysis established the prediction model in the training set. In the model, the score of each predictor was calculated. A nomogram made the model visualized. Thirdly, model discrimination was evaluated by receiver operating characteristic (ROC) curve analysis. The area under the curve (AUC) for 0.75 or more indicated good discrimination. The prediction accuracy was assessed by calibration plots. The clinical utility was estimated by decision curve analysis (DCA). The nomogram was validated by the test set. All tests were two-tailed tests and *p* ≤ 0.05 was statistically significant. We performed statistical analysis by STATA 15.0 (Stata Corporation, College Station, Texas, USA).

## Result

### Patient characteristics

Four hundred one eligible patients were finally recruited for the study. Participants’ characteristics were shown in Table 1. The average age was 62.26 + 14.23 years. 60.1% of patients were men. The hyperkalemia incidence was 39.65%. All enrolled patients were randomly divided into development (*n* = 256) and validation sets (*n* = 145) with a proportion of 6:4. There was no significant difference in clinical variables between development and validation cohorts (Table 2). The median (IQR) time of hemodialysis duration was 14 (7, 48) months in the development set and 16 (7, 48) months in the validation cohort. Hyperkalemia was observed in 102 (39.8%) patients in the training cohort and 57 (39.3%) in the validation set.

**Table 1:** Baseline characteristics of study patients with hemodialysis

Variable	HK (*n* = 159)	Non-HK (*n* = 242)	*P*-value
Age, year	61.86 ± 12.22	62.53 ± 15.43	0.631
Blood flow (mm/s)	245.53 ± 27.80	238.80 ± 37.49	0.040
Height(cm)	162.86 ± 8.13	162.39 ± 8.02	0.566
Pre-dialysis weight (kg)	61.08 ± 11.52	58.85 ± 12.02	0.065
Hemoglobin(g/L)	114.22 ± 20.31	104.85 ± 21.94	0.000
Serum sodium(mmol/L)	137.43 ± 3.66	138.84 ± 3.49	0.000
Serum calcium(mmol/L)	2.06 ± 0.46	2.14 ± 0.29	0.050
Albumin(g/L)	37.72 ± 5.97	35.26 ± 5.40	0.000
TC(mmol/L)	4.40 ± 1.27	4.31 ± 1.30	0.497
HDL(mmol/L)	0.99 ± 0.34	0.95 ± 0.36	0.318
Hemodialysis duration (month)	30 (11, 55)	10 (5, 31.25)	<0.001
Number of hemodialysis sessions	267 (104, 308)	92 (17, 245.5)	<0.001
Ultrafiltration volume(L)	3000 (2200, 3500)	2300 (1600, 3000)	<0.001
CRP(mg/L)	2.16 (1, 9.2)	5 (2, 20.05)	0.001
BUN (mmol/L)	24.1 (17.2, 29.5)	15.1 (9.18, 23.25)	<0.001
Uric acid(μmol/L)	382 (295, 469)	336.18 (185.75, 436.75)	0.001
eGFR(ml/min)	4.9 (3.9, 6.7)	7.25 (4.80, 12.30)	<0.001
Glucose(mmol/L)	5.87 (4.89, 8.16)	5.37 (4.59, 7.41)	0.031
Serum creatinine(μmol/L)	778 (614, 953)	581 (346, 804)	<0.001
Serum phosphate(mmol/L)	1.78 (1.31, 2.18)	1.37 (0.99, 1.79)	<0.001
TG(mmol/L)	1.32 (0.92, 2.03)	1.39 (0.96, 1.93)	0.985
LDL(mmol/L)	2.02 (1.48, 2.62)	2.03 (1.58, 2.56)	0.962
PTH(pg/ml)	238 (139.4, 445.6)	213.4 (82.33, 384.45)	0.020
Gender			0.292
Male	90 (56.6%)	151 (62.4%)	
Female	69 (43.4%)	91 (37.6%)	
Primary disease			0.309
Primary nephropathy	59 (37.11%)	107 (44.2%)	
Glomerulonephritis	33 (20.75%)	51 (21.07%)	
IgA nephropathy	20 (12.58%)	45 (18.59%)	
Membranous nephropathy	5 (3.14%)	8 (3.31%)	
Interstitial nephritis	1 (0.63%)	3 (1.24%)	
Secondary nephropathy	79 (49.69%)	98 (40.5%)	
Diabetes nephropathy	37 (23.27%)	43 (17.77%)	
Hypertensive nephropathy	42 (26.42%)	55 (22.73%)	
Hereditary nephropathy	3 (1.9%)	10 (4.1%)	
Unknown etiology	18 (11.32%)	27 (11.16%)	
Professional			0.693
Farmer	126 (79.7%)	189 (78.1%)	
Worker	4 (2.5%)	8 (3.3%)	
Clerk	8 (5.1%)	7 (2.9%)	
Retirees	7 (4.4%)	19 (7.9%)	
Student	0 (0.0%)	1 (0.4%)	
Self-employed	8 (5.1%)	11 (4.5%)	
Unemployed people	5 (3.2%)	7 (2.9%)	
Education level			0.501
Primary school diploma	32 (20.3%)	63 (26.0%)	
Junior high school diploma	26 (16.5%)	43 (17.8%)	
High school diploma	8 (5.1%)	15 (6.2%)	
College diploma	6 (3.8%)	11 (4.5%)	
Illiteracy	86 (54.4%)	110 (45.5%)	
Hemodialysis vascular access			0.007
Temporary tube	5 (3.1%)	15 (6.2%)	
Long term tube	36 (22.6%)	84 (34.7%)	
Fistula	118 (74.2%)	143 (59.1%)	
Dialysis modalities			0.300
HD	26 (16.4%)	51 (21.1%)	
HD + HF	133 (83.6%)	191 (78.9%)	
Furosemide use			0.076
Yes	118 (74.2%)	158 (65.3%)	
No	41 (25.8%)	84 (34.7%)	
ACEI/ARB			0.342
Yes	103 (64.8%)	169 (69.8%)	
No	56 (35.2%)	73 (30.2%)	
Hypertension			0.391
Yes	39 (24.5%)	50 (20.7%)	
No	120 (75.5%)	192 (79.3%)	
Diabetes mellitus			0.040
Yes	86 (54.1%)	157 (64.9%)	
No	73 (45.9%)	85 (35.1%)	

*HK* hyperkalemia, *TG* triglyceride, *HDL* high-density lipoprotein, *CRP* C-reactive protein, *BUN* blood urea nitrogen, *eGFR* estimated Glomerular Filtration Rate, *TC* total cholesterol, *LDL* low-density lipoprotein, *PTH* parathyroid hormone, *HD* hemodialysis, *HF* hemofiltration, *ACEI* angiotensin converting enzyme inhibitor, *ARB* angiotensin receptor blocker

Categorical variables are presented as n (%). Continuous variables with normal distribution are reported as mean ± SD. Continuous variables with abnormal distribution are given as median (IQR)

**Table 2:** The characteristics of development and validation sets

Variable	Development set (*n* = 256)	Validation set (*n* = 145)	*P*-value
Age, year	62.62 ± 14.71	61.64 ± 13.37	0.510
Blood flow (mm/s)	241.19 ± 34.80	241.97 ± 32.95	0.827
Height(cm)	162.32 ± 8.15	163.03 ± 7.91	0.399
Pre-dialysis weight (kg)	59.59 ± 12.03	59.99 ± 11.58	0.750
Hemoglobin(g/L)	107.79 ± 22.45	109.94 ± 20.52	0.340
Serum sodium(mmol/L)	138.36 ± 3.71	138.15 ± 3.47	0.575
Serum calcium(mmol/L)	2.13 ± 0.36	2.08 ± 0.38	0.223
Albumin(g/L)	36.43 ± 5.97	35.88 ± 5.35	0.353
TC(mmol/L)	4.35 ± 1.26	4.34 ± 1.34	0.913
HDL(mmol/L)	0.97 ± 0.36	0.96 ± 0.34	0.833
Hemodialysis duration (month)	14 (7, 48)	16 (7, 48)	0.871
Number of hemodialysis sessions	145.5 (36.5, 305)	137 (38, 306)	0.838
Ultrafiltration volume(L)	2500 (1800, 3200)	2700 (1882.50, 3300)	0.203
CRP(mg/L)	4.14 (1, 15.34)	3.17 (1.00, 12.20)	0.498
BUN (mmol/L)	18.1 (11.73, 27.05)	18.7 (10.70, 24.85)	0.681
Uric acid(μmol/L)	360 (246.5, 456)	353 (232, 424)	0.402
eGFR(ml/min)	5.90 (4.30, 9.50)	6.4 (4.30, 10.45)	0.761
Glucose(mmol/L)	5.48 (4.59, 7.42)	6.05 (4.90, 8.45)	0.051
Serum creatinine(μmol/L)	668 (452, 876.5)	675 (369.50, 893.50)	0.783
Serum phosphate(mmol/L)	1.5 (1.16, 1.97)	1.44 (1.05, 1.95)	0.381
TG(mmol/L)	1.36 (0.96, 1.91)	1.37 (0.93, 2.11)	0.777
LDL(mmol/L)	2.04 (1.60, 2.58)	1.98 (1.44, 2.57)	0.675
PTH(pg/ml)	239.60 (103.10, 427.98)	212.00 (101.65, 387.05)	0.163
Gender			0.617
Male	151 (59.0%)	90 (62.1%)	
Female	105 (41.0%)	55 (37.9%)	
Primary disease			0.235
Primary nephropathy	115 (44.9%)	51 (35.17%)	
Glomerulonephritis	60 (23.44%)	24 (16.55%)	
IgA nephropathy	47 (18.36%)	18 (12.41%)	
Membranous nephropathy	5 (1.95%)	8 (5.52%)	
Interstitial nephritis	3 (1.17%)	1 (0.69%)	
Secondary nephropathy	105 (41.01%)	72 (49.66%)	
Diabetes nephropathy	50 (19.53%)	30 (20.69%)	
Hypertensive nephropathy	55 (21.48%)	42 (28.97%)	
Hereditary nephropathy	7 (2.73%)	6 (4.14%)	
Unknown etiology	29 (11.33%)	16 (11.03%)	
Professional			0.636
Farmer	199 (78.0%)	116 (80.0%)	
Worker	8 (3.1%)	4 (2.8%)	
Clerk	11 (4.3%)	4 (2.8%)	
Retirees	18 (7.1%)	8 (5.5%)	
Student	1 (0.4%)	0 (0.0%)	
Self-employed	13 (5.1%)	6 (4.1%)	
Unemployed people	5 (2.0%)	7 (4.8%)	
Education level			0.653
Primary school diploma	58 (22.7%)	37 (25.5%)	
Junior high school diploma	47 (18.4%)	22 (15.2%)	
High school diploma	13 (5.1%)	10 (6.9%)	
College diploma	9 (3.5%)	8 (5.5%)	
Illiteracy	128 (50.2%)	68 (46.9%)	
Hemodialysis vascular access			0.588
Temporary tube	13 (5.1%)	7 (4.8%)	
Long term tube	81 (31.6%)	39 (26.9%)	
Fistula	162 (63.3%)	99 (68.3%)	
Dialysis modalities			1.000
HD	49 (19.1%)	28 (19.3%)	
HD + HF	207 (80.9%)	117 (80.7%)	
Hyperkalemia			1.000
Yes	154 (60.2%)	88 (60.7%)	
No	102 (39.8%)	57 (39.3%)	
Furosemide			0.101
Yes	184 (71.9%)	92 (63.4%)	
No	72 (28.1%)	53 (36.6%)	
ACEI/ARB			0.280
Yes	179 (79.9%)	93 (64.1%)	
No	77 (30.1%)	52 (35.9%)	
Hypertension			0.804
Yes	58 (22.7%)	31 (21.4%)	
No	198 (77.3%)	114 (78.6%)	
Diabetes mellitus			0.254
Yes	161 (62.9%)	82 (56.6%)	
No	95 (37.1%)	63 (43.4%)	

*HK* hyperkalemia, *TG* triglyceride, *HDL* high-density lipoprotein, *CRP* C-reactive protein, *BUN* blood urea nitrogen, *eGFR* estimated Glomerular Filtration Rate, *TC* total cholesterol, *LDL* low-density lipoprotein, *PTH* parathyroid hormone, *HD* hemodialysis, *HF* hemofiltration, *ACEI* angiotensin-converting enzyme inhibitor, *ARB* angiotensin receptor blocker

Categorical variables are presented as n (%). Continuous variables with normal distribution are reported as mean + SD. Continuous variables with abnormal distribution are given as median (IQR)

### Predictors of hyperkalemia

**Fig. 1:**
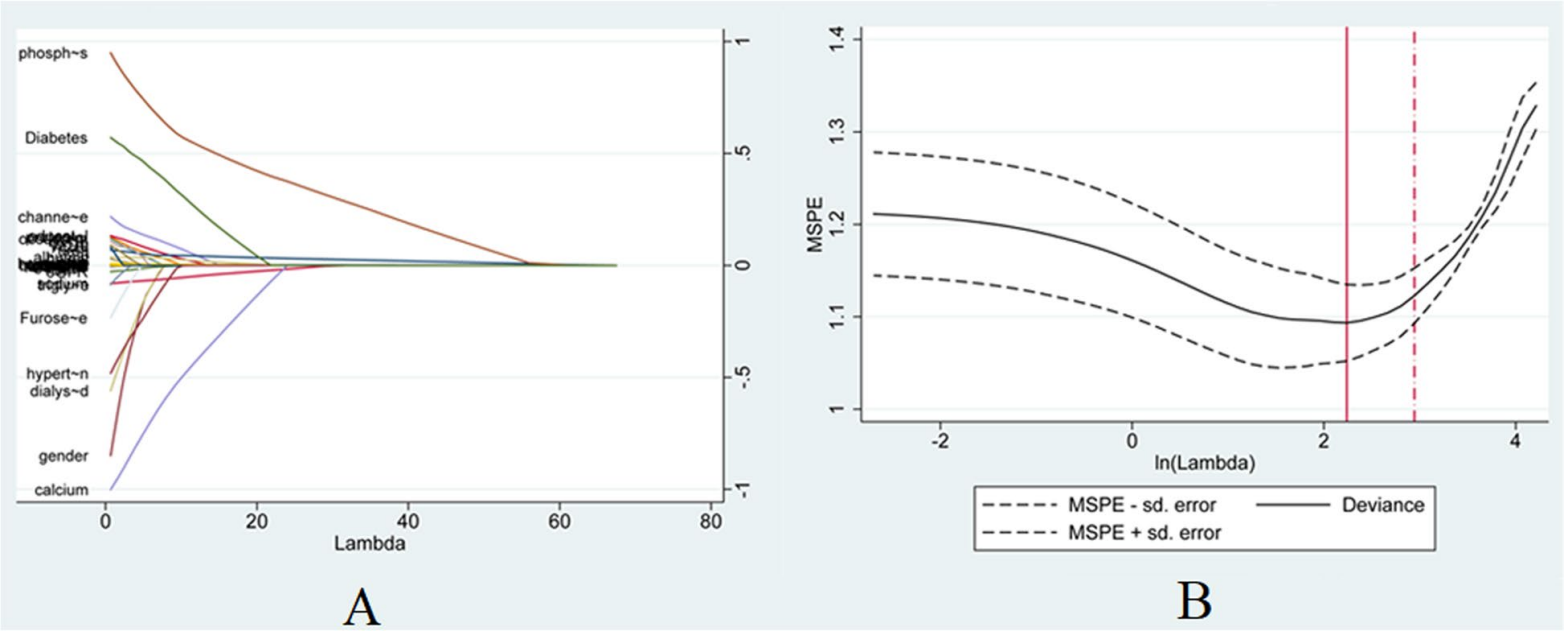
LASSO coefficient profiles of hyperkalemia. **A** Each curve in the figure presents the change of each variable in coefficient. The ordinate is the coefficient value, the lower abscissa is log(λ), and the upper abscissa is the number of non-zero coefficients in the model at this time. **B** 10-fold cross-cross validation fitting and then selecting the model

Firstly, we preliminarily selected predictors of hyperkalemia by LASSO regression. Variables were centralized and normalized by 10-fold cross-validation (Fig. 1). Selected predictors were the number of hemodialysis sessions, ultrafiltration volume, hemoglobin, BUN, serum sodium, serum calcium, albumin, serum phosphorus, and diabetes. Secondly, we included six predictors as independent risk variables to construct a prediction model by multivariable logistic regression. These six predictors were the number of hemodialysis sessions (OR: 1.01; 95%CI: 1.00–1.01), BUN (1.05; 1.01–1.08), serum sodium (0.90; 0.83–0.98), serum calcium (0.37; 0.16–0.85), serum phosphorus (2.54; 1.47–4.37), and diabetes (2.37; 1.25–4.46) (Table 3).

**Table 3:** Multivariate logistic regression analysis of predictors selected by LASSO regression procedure in the development set

Independent variables	Multivariable logistic regression analysis	
	OR (95% CI)	*P*-value
Number of hemodialysis sessions	1.007 (1.004–1.010)	< 0.001
BUN	1.047 (1.014–1.082)	0.006
Serum sodium	0.900 (0.825–0.982)	0.018
Serum calcium	0.374 (0.164–0.852)	0.019
Serum phosphorus	2.535 (1.469–4.374)	0.001
Diabetes	2.365 (1.253–4.463)	0.008

*BUN* blood urea nitrogen

### Nomogram of hyperkalemia in patients with hemodialysis

**Fig. 2:**
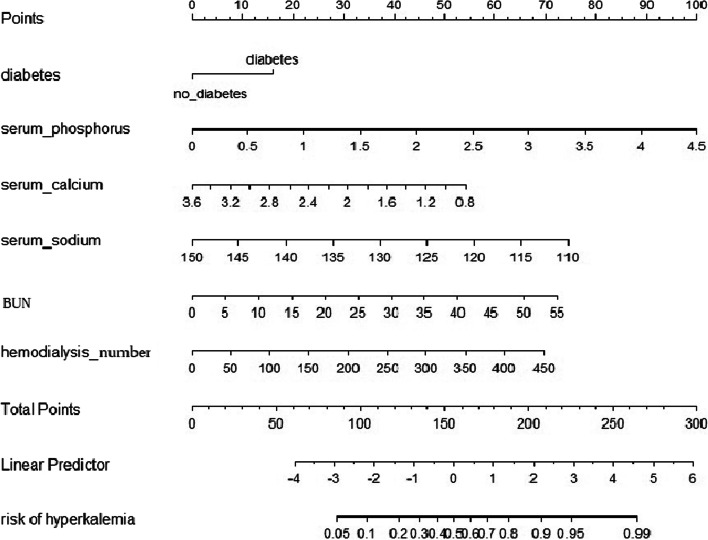
The nomogram for predicting the risk of hyperkalemia in patients with hemodialysis. Each level of predictor indicates a certain score. A total score was generated by a summary of the score of each predictor. The total score corresponds to hyperkalemia probability. BUN: blood urea nitrogen

A nomogram was constructed to predict the risk of hyperkalemia in patients with hemodialysis. This model contained six predictors: number of hemodialysis sessions, BUN, serum sodium, serum calcium, serum phosphorus, and diabetes. For example, a 60-years-old man with diabetes accepted hemodialysis treatment three times per week for 4 months. He experienced 48 times of hemodialysis. His blood laboratory tests in the last pre-hemodialysis were BUN 25 mmol/L, serum sodium 140 mmol/L, serum calcium 2.8 mmol/L, and serum phosphorus 2 mmol/L. The corresponding score of each predictor was 15 points, 8 points, 35 points, 20 points, 15 points, and 45 points respectively. His total score was 138 points. It indicated that the risk of hyperkalemia was 35% in this patient (Fig. 2).

### Evaluation and validation of the nomogram

**Fig. 3:**
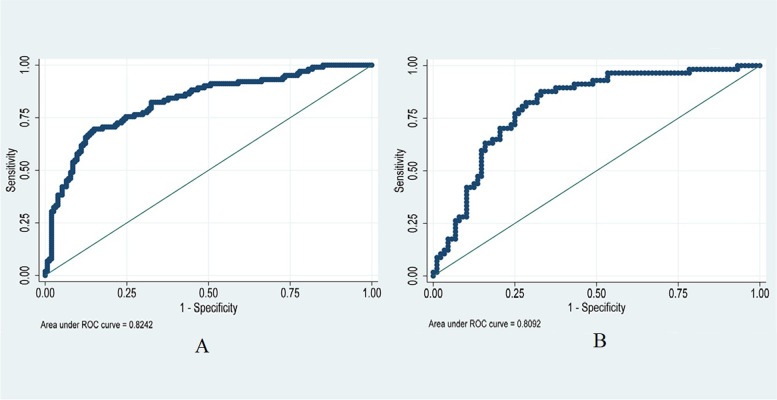
ROC curve and AUC of the predictive model. **A** The ROC in the development set. **B** The ROC in the validation set. ROC: receiver operating characteristic; AUC: area under the curve

**Fig. 4:**
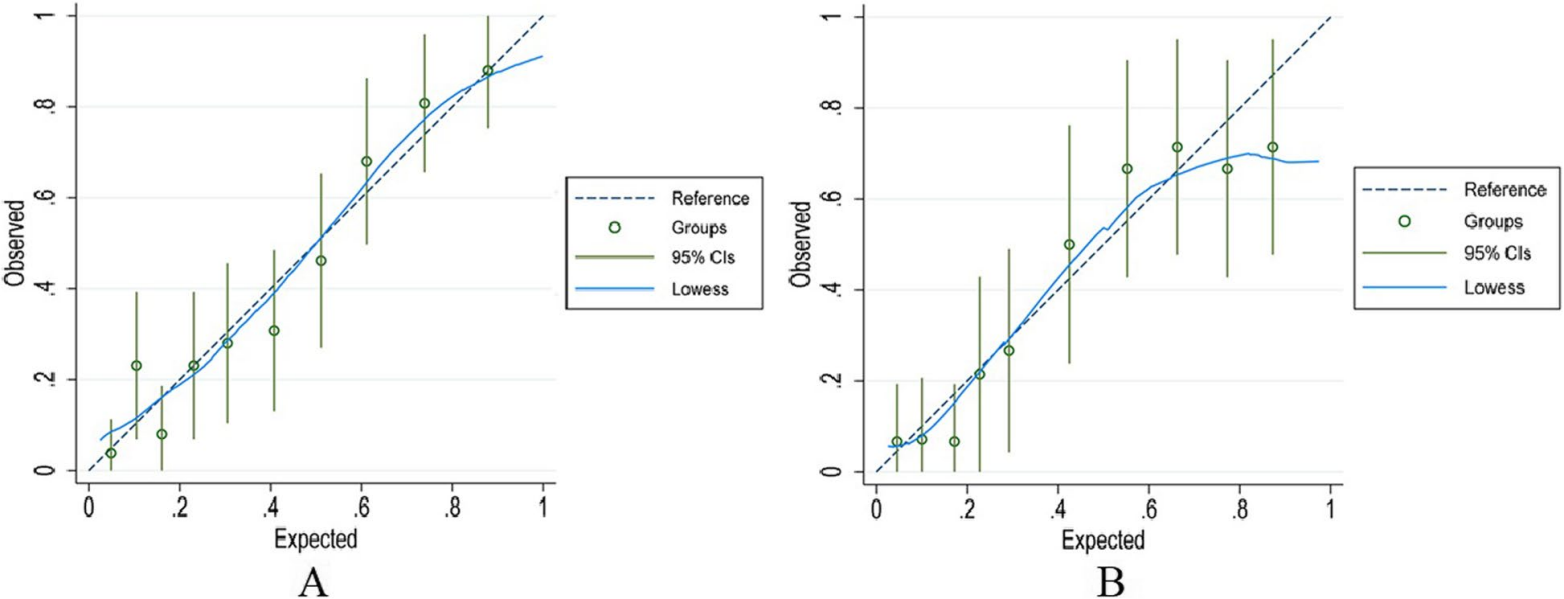
Calibration plots of the predictive model. **A** Calibration plot in the development set. **B** Calibration plot in the validation set. The x-axis is the predicted probability of hyperkalemia. The y-axis is the observed hyperkalemia. The diagonal dotted line represents a perfect prediction by an ideal model. The solid line represents the performance of the nomogram. It represents a better prediction that a solid line is close to a diagonal dotted line. The figure shows that the prediction model has a good predictive ability

**Fig. 5:**
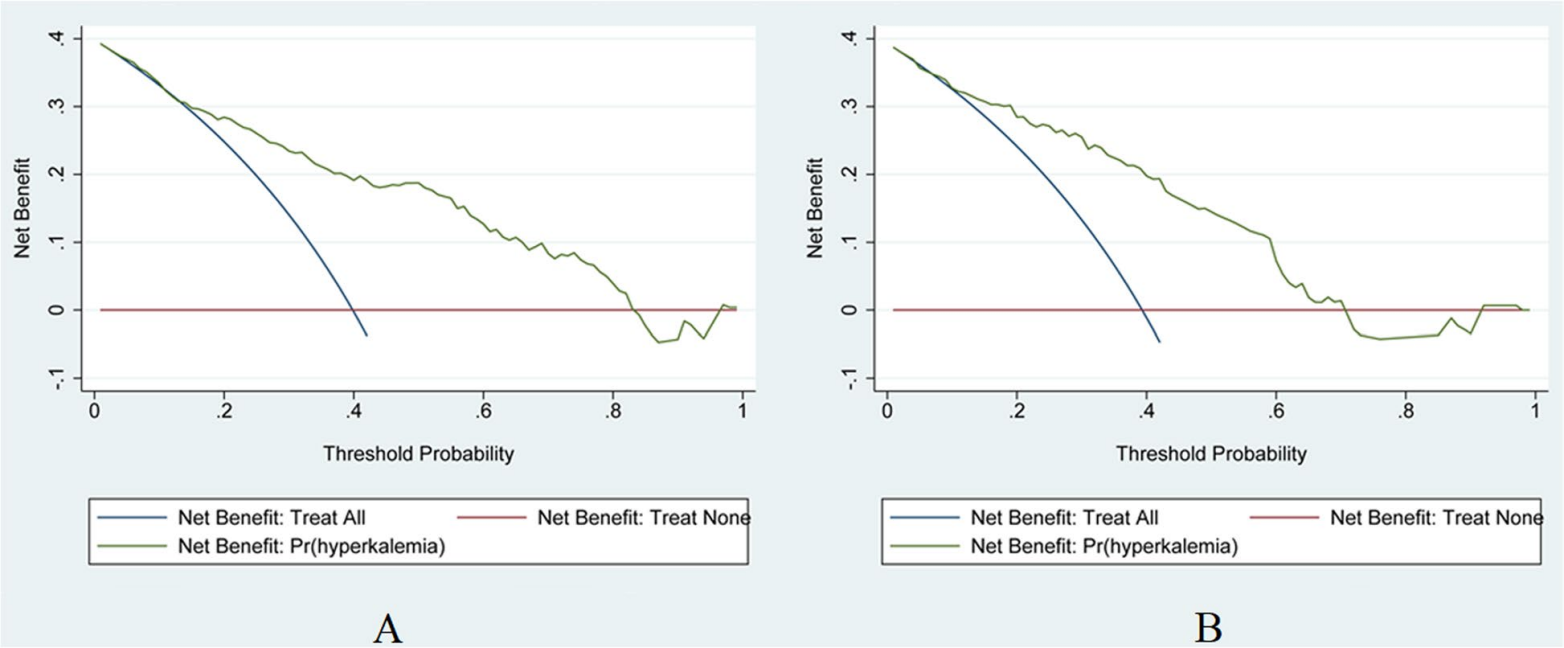
DCA of the nomogram. **A** DCA in the development set. **B** DCA in the validation set. Red-solid line: The patient does not apply the nomogram and the net benefit is zero; Blue-solid line: All patients are treated by the nomogram. The area enclosed by the three lines presents the clinical utility of the nomogram. DCA: decision curve analysis

On the Hosmer-Lemeshow test, the development set was χ^2^ = 8.20, *p* = 0.61 and the validation set was χ^2^ = 6.92, *p* = 0.73. This result presented that the predicted outcomes were close to the observed outcomes. The ROC curve in training set showed a good discrimination (AUC: 0.82; 95% CI: 0.77–0.88) (Fig. 3A). The discrimination performance of the model was validated in the test set (0.81; 0.74–0.88) (Fig. 3B). Furthermore, calibration curve analysis demonstrated a good concordance between the predicted probabilities and the observed hyperkalemia in the training and test sets (Fig. 4). DCA exhibited this model with clinical utility (Fig. 5).

## Discussion

Previous studies developed models to predict the risk of hyperkalemia in patients with chronic kidney disease (CKD) [22, 23]. However, these models are suitable to predict CKD patients not dialysis-dependent (NDD) rather than dialysis-dependent (DD) patients. CKD is divided into five stages according to eGFR. CKD-NDD patients have a higher excretory function of kidney than DD patients. The clearance mostly relies on dialysis modality in CKD-DD patients. In patients with peritoneal dialysis, peritoneal dialysis performs a promising ability of potassium clearance. Therefore, they have a lower incidence of hyperkalemia and are more likely to occur with hypokalemia [24]. However, hemodialysis patients are usually confronted with hyperkalemia. Therefore, it is vital to establish a model for predicting hyperkalemia in hemodialysis populations.

In this study, the incidence of hyperkalemia was 39.65% in patients with hemodialysis. It was consistent with previous studies [6, 25]. This study firstly developed a model for hyperkalemia prediction in patients with hemodialysis. Predictors in this model were the number of hemodialysis sessions, BUN, serum sodium, serum calcium, serum phosphorus, and diabetes. This nomogram was applied as a guide for clinicians to predict the risk of hyperkalemia.

Several studies investigated the relationship between hyperkalemia and mortality, or cardiovascular disease. They showed hyperkalemia is increasingly becoming one of the complications to increase mortality rate in patients with hemodialysis [26]. A serum potassium concentration of more than 5.6 mmol/L increases the risk of all-cause and cardiovascular death [27]. However, the risk factors of hyperkalemia are poorly reported. Consistent with previous analyses [28–30], this study supported diabetes as a risk factor for hyperkalemia. Insulin pumps Na^+^ out and K^+^ into the cell by Na^+^ − K^+^ ATPase. Insulin deficiency reduces the cellular intake of potassium and leads to more potassium on the outside of the cell. In patients with ESRD, uremia decreases tissue sensitivity to insulin. Diabetes burdens the accumulation of extracellular potassium. Consequently, diabetes can be a predictor of hyperkalemia.

In patients with hemodialysis, a large portion of BUN is cleared from the blood by hemodialysis modality. The adequacy of hemodialysis is evaluated by the removal of BUN clinically [31]. Greater levels of BUN indicate poor adequacy of hemodialysis. The poor adequacy of hemodialysis reduces the clearance of serum potassium and phosphorus. This study discovered that the levels of BUN and serum phosphorus were positively correlated with hyperkalemia. This finding is supported by a previous report. The report concluded a significant relationship between serum potassium and BUN (*p* < 0.01) [28]. Therefore, higher levels of BUN and serum phosphorus were demonstrated as risk predictors of hyperkalemia in patients with hemodialysis. On the contary, the levels of serum sodium and calcium were discovered to negatively interact with hyperkalemia. The OR values of serum sodium and calcium are less than 1. It presents that lower levels of serum sodium and calcium indicate a higher probability of hyperkalemia. This negative association probably results from the work of Na^+^ − K^+^ ATPase [32]. Na^+^ − K^+^ ATPase exchanges 3 cytoplasmic Na^+^ for every 2 extracellular K+ that move into the cell. During phase 2 and 3 of the cardiac action, K^+^ efflux and Na^+^/Ca^2+^ influx complete repolarization [33]. This antiporter explains the negative correction between K^+^ and Na^+^/Ca^2+^.

It is difficult for clinicians to precisely calculate the level of potassium intake from diet.Therefore, this study didn’t include dietary potassium intake in the model. Furthermore, it is still controversial whether dietary potassium intake influences serum potassium and induces hyperkalemia in patients with hemodialysis. Some studies reported that potassium intake more than excretion enhances the risk of hyperkalemia. However, in these studies, the doses of potassium supplements largely exceed the recommended level of dietary potassium intake. Although the daily potassium intake may surpass the extracellular content, the daily change in serum potassium level is less than 10% [33]. Noori et al. found dietary potassium intake was weakly correlated (r = 0.14) with serum potassium concentrations [31]. Ramos et al. assessed dietary potassium intake in 95 patients with CKD-NDD and 117 patients with hemodialysis [27]. They didn’t discover a significant association between serum potassium and dietary potassium intake. They also supported previous cross-sectional studies that dietary potassium explained less than 2% of the variation in serum potassium. These reports suggested potassium intake doesn’t determine the serum potassium in hemodialysis populations [30, 31, 34]. Recently, a Kidney Disease Improving Global Outcomes (KDIGO) conference found a limitation of direct evidence on a relationship between dietary potassium intake and serum potassium concentrations. KDIGO suggested that an optimal recommendation for dietary potassium intake should be investigated [35]. In addition, some evidence supports the health benefits of a potassium-rich diet including decreased risks of CKD progression, cardiovascular disease, and stroke [36]. Therefore, we suppose that dietary potassium intake should be viewed as an educational content rather than a predictor. Clinicians should recommend an optimal level of dietary potassium intake and pay attention to dietary education for patients.

RAASi is a kind of medication for hypertension, including angiotensin-converting enzyme inhibitors (ACEI) and angiotensin receptor blockers (ARB). They improve the outcomes for patients by decreasing cardiovascular events [37]. A lot of evidence supports the maximal administration of RAASi if patients tolerated it. However, hyperkalemia is a common adverse effect of RAASi. This adverse effect limits the use in patients with hemodialysis. A survey reported about 88% of suspending administration due to a serum potassium increase. This prediction model helps clinicians to identify the risk of hyperkalemia and decide whether RAASi should be continually prescribed. Additionally, with the introduction of novel potassium binders such as sodium zirconium cyclosilicate, clinicians predict patients with a requirement of novel potassium binders according to the model.

### Study limitation

There were some limitations to this study. Firstly, we haven’t externally validated the nomogram. We will continually recruit patients to complete the external validation. Secondly, we ignored the impact of metabolic acidosis on hyperkalemia. In the future, we will research the interaction between metabolic acidosis and hyperkalemia in the nomogram. Thirdly, we didn’t analyze the relationship between the dose of dietary potassium intake and hyperkalemia. It is necessary to investigate the daily optimal dose of dietary potassium intake.

## Conclusion

We established a risk prediction model of hyperkalemia for patients with hemodialysis. The risk model provide valuable insights into the identification of hyperkalemia risk and prevention.

## Data Availability

The data analyzed during this study are included in this published article.

## Abbreviations

ESRD: end-stage renal disease
LASSO: Least Absolute Shrinkage Selection Operator
ROC: receiver operating characteristic
DCA: decision curve analysis
BUN: blood urea nitrogen
KRT: kidney replacement therapy
AUC: area under the curve
ACEI: angiotensin-converting enzyme inhibitors
ARB: angiotensin receptor blockers
OR: odds ratio
95%CIs: 95% confidence intervals
eGFR: estimated glomerular filtration rate
LDL: low-density lipoprotein
HDL: high-density lipoprotein
PTH: parathyroid hormone
RAASi: renin-angiotensin-aldosterone system inhibitors
IQR: interquartile range
CKD: chronic kidney disease
